# Toward a hyperventilation detection system in freediving: a proof of concept using force sensor technology

**DOI:** 10.3389/fphys.2024.1498399

**Published:** 2025-01-06

**Authors:** Frank Pernett, Eric Mulder, Filip Johansson, Arne Sieber, Ricardo Bermudez, Marcus Lossner, Erika Schagatay

**Affiliations:** ^1^ Environmental Physiology Group, Department of Health Sciences, Mid Sweden University, Östersund, Sweden; ^2^ Swedish Winter Sports Research Centre, Department of Health Sciences, Mid Sweden University, Östersund, Sweden; ^3^ Oxygen Scientific GmbH, Graz, Austria; ^4^ Sensing Systems Corporation, Dartmouth, United States; ^5^ Independent hardware and software engineer, Atlanta, GA, United States

**Keywords:** tidal volume, breath-hold, apnea, blackout, wearable technology

## Abstract

**Background and aim:**

Hyperventilation before breath-hold diving (freediving) is widely accepted as a risk factor for hypoxic syncope or blackout (BO), but there is no practical way to address it before dives. This study explores the feasibility of using a force sensor to predict end-tidal carbon dioxide (*P*_ET_CO_2_) to assess hyperventilation in freedivers.

**Methods and results:**

Twenty-one freedivers volunteered to participate during two national competitions. The divers were instructed to breathe normally and perform three dry apneas of 1, 2, and 3-min duration at 2-min intervals in a sitting position. Before and after the apneas, *P*_ET_CO_2_ was recorded. The signal from the force sensor, attached to a chest belt, was used to record the frequency and amplitude of the chest movements, and the product of these values in the 60 s before the apnea was used to predict *P*_ET_CO_2_. The mean *P*_ET_CO_2_ was below 35 mmHg before all apneas. The mean amplitude of the signal from the force sensor increased from apnea 1 to apnea 3 (p < 0.001), while the respiratory rate was similar (NS). The product of the respiratory rate and amplitude from the force sensor explained 34% of the variability of the *P*_ET_CO_2_ in the third apnea.

**Conclusion:**

This study shows that a force sensor can estimate hyperventilation before static apnea, providing a basis for further research. More studies are needed to confirm its effectiveness in preventing issues. Freedivers may hyperventilate without noticing it, and such a system could improve awareness of this condition. Additional underwater tests are essential to determine whether this system can enhance safety in freediving.

## Introduction

Breath-hold divers, also referred to as freedivers, often employ a breathing pattern known as hyperventilation to extend their apnea duration by reducing the alveolar carbon dioxide pressure (PACO_2_). Volitional hyperventilation is a conscious effort to increase the breathing rate and depth, which increases alveolar ventilation, leading to a slight increase in alveolar oxygen pressure (PAO_2_), a reduction in PACO_2,_ lowering of arterial CO_2_ levels (hypocapnia), and an elevation in pH (West and Luks, 2021). In contrast, metabolism-driven hyperventilation is an automatic, homeostatic response to increased metabolic activity, such as during exercise, to expel excess CO_2_ and stabilize blood gas levels (Forster et al., 2012). Hyperventilation before breath-hold diving, despite a slight increase in arterial oxygen pressure (PaO_2_), leads to a greater risk of losing consciousness underwater (Craig, 1961; 1976; Edmonds and Walker, 1999; Lippmann and Pearn, 2012), as the control of ventilation relies on chemoreceptors that respond to changes in PaCO_2_ and hydrogen ion (H^+^) levels. As PaCO_2_ decreases due to hyperventilation, the ventilatory drive is compromised, leading to a delayed urge to breathe. Consequently, this results in an extended apnea duration (Hill, 1973; Lin et al., 1974; Bain et al., 2017; Pernett et al., 2023). A longer apnea duration increases the level of hypoxia during a breath-hold, which exposes the freediver to an increased risk of losing consciousness underwater, known as blackout (BO) or hypoxic syncope (Lindholm and Gennser, 2005; Kumar and Ng, 2010). The risk of severe oxygen desaturation has recently been found to be exacerbated during repeated series of apnea after short-time hyperventilation of 15 s (Pernett et al., 2023).

Hyperventilation is reported as a risky practice before breath-holding among recreational swimmers (Boyd et al., 2015), spearfishers (Lippmann, 2019), and snorkelers (Dunne et al., 2021). This breathing pattern increases apnea duration and desaturation, increasing the risk of BO, with the potential consequence of drowning if not promptly addressed. In addition, hyperventilation can reduce cerebral blood flow by 2% for each 1 mmHg of decline in PaCO_2_ (Raichle and Plum, 1972). Experienced freedivers exhibit enhanced tolerance to hypoxia as training seems to diminish their hypoxic ventilatory response (Schneeberger et al., 1986; Ferretti et al., 1991; Lindholm and Lundgren, 2006). This suggests that trained freedivers, when engaging in hyperventilation, may experience pronounced hypoxemia since they depend on the hypoxic stimulus to terminate the breath-hold.

Despite the evidence contradicting the benefits of hyperventilation, there remains a significant gap in knowledge concerning the prevalence and role of this practice among competitive freedivers, snorkelers, and spearfishers. Some insights into this issue have emerged from blood gas analyses in studies characterized by relatively modest sample sizes. In elite freedivers, documented pre-diving PaCO_2_ levels vary, with reported values of 29 mmHg (Molchanova et al., 2020), 26 mmHg (Muth et al., 2003), and 21 mmHg (Scott et al., 2021). In contrast, non-elite breath-hold divers and Ama divers exhibit pre-diving values within the normal range, registering PaCO_2_ levels of 38 ± 3 mmHg (mean ± SD; Bosco et al., 2018) and 42 ± 2 mmHg (mean ± SD; Qvist et al., 1993), respectively. However, evaluating pre-apnea PaCO_2_ or *P*_ET_CO_2_ in non-laboratory settings, during diving, poses practical challenges. Blood gas analysis, while providing precise data, demands specific expertise and is invasive. Similarly, measuring exhaled gases requires equipment susceptible to damage in aquatic environments. An alternative strategy involves the measurement of tidal volume (Vt) and respiratory rate (RR) to estimate the minute ventilation at rest.

Various studies have explored methods employing stretch, piezoelectric, optical, pressure, electromagnetic, or acoustic sensors; accelerometers; and electrical impedance techniques for estimating Vt and RR (Panahi et al., 2020; Monaco and Stefanini, 2021). These techniques essentially aim to monitor alterations in thoracic and abdominal movements to infer Vt. However, applying these sensors in the underwater environment is complex. Our laboratory has constructed a unique underwater monitor involving a force sensor in a buckle attached to a chest belt, allowing detailed chest movement recording (Sieber et al., 2022). The main goal of the current study was to determine whether respiratory data obtained using this custom-built force sensor could predict *P*_ET_CO_2_ before a static breath-hold to assess the practice of hyperventilation in freedivers. Additionally, the secondary aim was to visually assess the signal quality when the sensor was used on divers in the pool.

## Methods

### Device description

**FIGURE 1:**
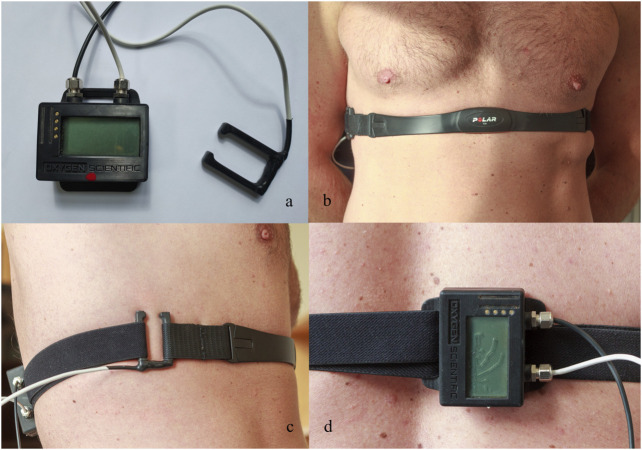
Custom-made buckle equipped with four strain gauges aligned on the sensor to measure the strain created by the applied force along with the data logger **(A)**. Frontal **(B)** and lateral **(C)** view of the buckle in its operational position attached to the chest strap. Details of the data logger in operational position on the back of the participant **(D)**.

The concept involved designing a U-shaped buckle (Figure 1A) that is both water- and pressure-proof and equipped with integrated strain gauges. These gauges enable the detection of pulling forces exerted on the buckle, facilitating the monitoring of alterations in chest circumference during underwater activities (Figure 1C). The description of this force sensor has been previously documented (Sieber et al., 2022). The custom buckle was crafted through a conventional biomechanical engineering design process employing machine drawing techniques. The choice of stainless steel as the buckle material was made to ensure its suitability for use in saltwater environments. The sensor was calibrated by applying a force of 10 N to one of its legs, producing a slight bending of the section connecting the two legs (Figure 1A). The applied force was correlated with the electrical signal from the sensor. To ensure water resistance, the entire buckle, including the strain gauges, was coated with a multipurpose rubber coating (Plasti Dip International, Blaine, MN). The strap from a commercially available Polar heart rate belt (Polar T34, Polar Electro, OY, Finland) was used to place the buckle on the chest (Figure 1B).

An improved version of a data logger, constructed by our laboratory previously, was used to read out the signals of the sensor-equipped buckle (Mulder et al., 2021; Figures 1A, D). Due to the low amplitude of the signals of the sensor-equipped buckle, it was necessary to employ an amplifier and a high-resolution analog-to-digital converter. We opted for the AD7192 analog-to-digital converter by Analog Devices, which is specifically designed for strain gauge signal acquisition. This integrated circuit combines a programmable gate array with a maximum 128x amplification, a 24-bit sigma–delta ADC, and a filtering stage, which effectively suppresses noise, particularly from 50 or 60 Hz power lines. Further improvements to the data logger included a USB port, a Bluetooth module, a digital pressure sensor, and a 3 × 16 character LC display.

### Vital capacity calibration

The calibration procedure aimed to test the accuracy of the force sensor to estimate the vital capacity (VC). Details about the VC calibration are presented in Supplementary Material. The equation for the predicted VC was VC = 1.6 + (0.4396 × amplitude). The difference between the measured VC and the predicted VC was 0.00 ± 0.7 L (Supplementary Figure S1B).

### Participants

The study included 21 participants (5 female and 16 male) with a mean ± SD age of 44 ± 7 years, a height of 178 ± 9 cm, a weight of 73 ± 10 kg, and a lung vital capacity of 5.82 ± 1.22 L. All participants were trained freedivers. Their training load was 5 ± 7 h per week. The study was conducted during two freediving national competitions. The divers competed in four pool disciplines. The participants received written and oral information on the protocol, after which they signed an informed consent document. The protocol was approved by the Swedish Research Ethics Authorities (EPM; #2019-05147) and complied with the Helsinki Declaration of 2004, apart from preregistration in a database.

### Study design

**FIGURE 2:**
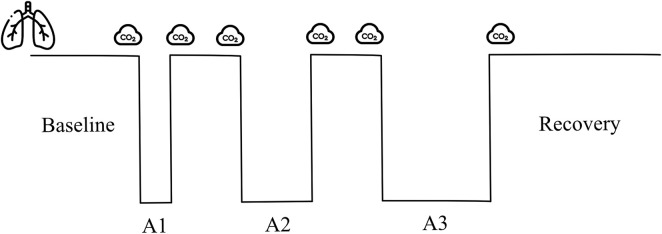
Apnea test protocol, involving apneas of 1, 2, and 3 min duration (A1–A3). Icons represent the time of vital capacity and exhaled CO_2_ measurements. Baseline (3-min). Between apnea breathing intervals (2-min). Recovery (5-min).

The study involved a dry static apnea ramp test with durations of 1, 2, and 3 min, respectively, spaced by 2 min of recovery (Figure 2).

### Procedures

A field laboratory was setup within the same pool area where the competitions took place. Participants were required to have a minimum of 12 h of rest following maximal performance and at least 2 h of fasting before initiating the test. Height, weight, and slow vital capacity were measured in triplicate in standing conditions, and the largest volume was used (Compact Expert, Vitalograph, Buckingham, United Kingdom). The participants filled out a questionnaire with information on the training load and personal best achievements in different freediving disciplines in the last 12 months. The participants performed a series of three apneas with fixed duration in dry conditions and the seated position (Figure 2). A researcher carried out a 2-min countdown before starting. At 30 s before apnea, a nose clip was applied, and 20 s before apnea, a mouthpiece was offered to breathe through. Ten seconds before the apnea, the countdown continued second by second.

Participants were instructed to exhale completely and then take a large, but not maximal, inhalation before starting the apnea voluntarily; this technique results in a volume of approximately 80 - 85% of the vital capacity (Schagatay and Holm, 1996). The participants were instructed to avoid hyperventilation. An experimenter closely monitored peripheral arterial oxygen saturation S_p_O_2_ and was ready to interrupt the apnea should it fall below 65%. The room temperature was 26.9°C ± 2.0°C.

### Measurements

*P*_ET_CO_2_ was measured before and after every apnea via an infrared-based gas measurement module (LifeSense LS1-9R, Nonin Medical Inc., Plymouth, United States). The diver breathed through a disposable mouthpiece with a bacterial filter connected to a T-valve with two one-way valves (AFT21, Biopac Systems, Goleta, United States). S_p_O_2_ and heart rate (HR) were measured using a reflectance sensor (800R, Nonin Medical Inc., Plymouth, United States) placed on the forehead 1 cm over the left eye and connected to a clinical monitor (LifeSense, Nonin Medical Inc., Plymouth, United States). Breathing movements were measured continuously using the prototype force sensor (Sieber et al., 2022).

Six male freedivers were also outfitted with the prototype force sensor prior to engaging in their freediving competition performances. This was done to assess the signal quality in the underwater environment.

### Data analysis

**FIGURE 3:**
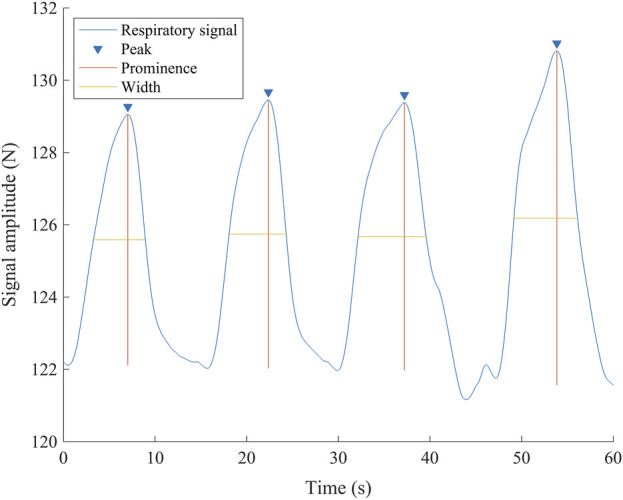
Representative respiratory signal from one participant 60 s before the breath-hold. The inverted triangles show the detected peaks of the signal, corresponding to the end of inhalation, and are used to calculate the respiratory rate. The vertical lines show the amplitude of every wave (prominence) used as a surrogate of tidal volume. The horizontal lines show the duration measured at the midpoint of the prominence (width). N, newton; s, seconds.

*P*_ET_CO_2_ used was the highest value measured after the last exhalation before the apnea. The data from the data logger were extracted and analyzed with custom-made scripts using MATLAB (R2022b, MathWorks Inc., Natick, United States). Within the final minute leading up to the last exhalation before commencing the breath-hold, both breathing frequency and signal amplitude were extracted for analysis. The number of peaks in the respiratory signal represented the RR, and the prominence of the signal was used as the surrogate of Vt. With those values, the estimated minute ventilation (eMv) was calculated as the product of the RR and the amplitude of the prominence (RR x prominence; Figure 3).

### Statistical analysis

The statistical analysis was carried out using SPSS 27 software (IBM Corp, Armonk, United States). The data were tested for normality using Shapiro–Wilk test and are reported as the mean ± SD. Outliers were defined as cases with a studentized deleted residual greater than three standard deviations (SD). A Spearman’s correlation test was run to assess the relationship between VC and *P*_ET_CO_2_ and the amplitude of the signal from the force sensor. A one-way repeated measures analysis of variance (ANOVA) was used to compare VC and amplitude of the respiratory signal and compare *P*_ET_CO_2_, amplitude, and eMv before every apnea. The Bonferroni correction for multiple comparisons was applied. Significance was observed at p < 0.05. A linear regression was run to predict *P*E_T_CO_2_ from the eMV before every apnea. The Bland–Altman method was used to assess the agreement between the measured VC and predicted VC and between measured *P*E_T_CO_2_ and predicted *P*E_T_CO_2_ (Bland and Altman, 1986). The accepted clinical limits of agreement (LOA) for capnography are ≤5 mmHg (Wu et al., 2003), but the LOA between *P*E_T_CO_2_ and PaCO_2_ could be as large as + 31 mmHg (İşat et al., 2023). When comparing two methods for measuring *P*E_T_CO_2_, the LOA could be 11 mmHg (Tamashiro et al., 2023). We set the accepted LOA to ≤10 mmHg. Effect sizes were estimated by the partial eta squared ( $\eta_{p}^{2}$ ) and the generalized eta squared ( $\eta_{G}^{2}$ ) and are presented with a 90% confidence interval (CI). An effect size of 0.01–0.05 was considered small, 0.06–0.13 was considered medium, and 0.14 and above was considered large (Cohen, 1988; Bakeman, 2005; Lakens, 2013).

End-tidal carbon dioxide (*P*_ET_CO_2_) data before the first apnea were missing for one participant; therefore, analyses for apnea 1 were conducted with data from 20 participants, as indicated in the results.

## Results

All participants completed the apnea protocol as intended, except four participants, who were unable to reach the full 3-min duration during the third apnea. These divers were included in the analysis, resulting in an average duration of 174 ± 13 s for A3.

### Respiratory values

*P*_ET_CO_2_ was lower before the last apnea (A3) than before the first apnea (A1, p = 0.034, $\eta_{p}^{2}$ = 0.16, 90% CI [0.01–0.31], and $\eta_{G}^{2}$ = 0.03; Table 1). The RR was lower in A2 than in A1 (p = 0.034, $\eta_{p}^{2}$ = 0.20, 90% CI [0.02–0.34], and $\eta_{G}^{2}$ = 0.05; Table 1).

**TABLE 1:** Pre-apnea respiratory values and data from respiratory buckle signal.

	*P*_ET_CO_2_ (mmHg)		RR (bpm)	Amplitude (N)	Vt (L)	eMv (N/min)
	Measured	Predicted				
A1^#^	31 ± 5	31 ± 3	10 ± 3	2.9 ± 1.8	2.87 ± 2.39	25.6 ± 12.6
A2	31 ± 7	33 ± 4	8 ± 2*	3.7 ± 2.5^*^	3.23 ± 2.70	28.7 ± 16.1
A3	29 ± 6*	30 ± 4	9 ± 3	4.0 ± 2.4*	3.36 ± 2.66	32.4 ± 14.1*

Values are presented as the mean ±1 SD. #n = 20. *P*_ET_CO_2,_ end-tidal exhaled pressure of carbon dioxide; RR, respiratory rate; bpm, breaths per minute; N, newton; Vt, calculated tidal volume; L, liters, eMV, estimated minute ventilation calculated as the product of the RR and the amplitude of the prominence in the respiratory signal; A1–A3, apnea 1 to apnea 3. *Significantly different from A1.

### Respiratory signal

The signal from the device was clear, and RR and amplitude were easily detectable (Figure 3). The amplitude was larger in A2 and A3 than in A1 (p < 0.001, $\eta_{p}^{2}$ = 0.36, 90% CI [0.12–0.52], and $\eta_{G}^{2}$ = 0.05; Table 1). The product of the amplitude and the respiratory rate (eMV) was higher in A3 than in A1 (p < 0.001, $\eta_{p}^{2}$ = 0.35, 90% CI [0.14–0.49], and $\eta_{G}^{2}$ = 0.04; Table 1).

### Correlation analysis of estimated tidal volume with *P*_ET_CO2

**FIGURE 4:**
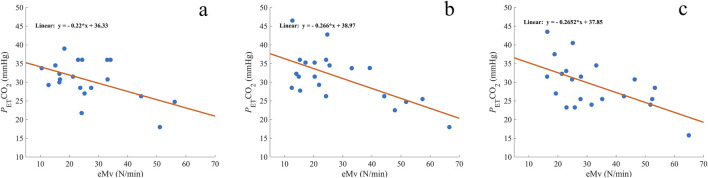
Comparison of measured end-tidal CO_2_ (*P*_ET_CO_2_) with the estimated minute ventilation (eMV) before A1 **(A)**, A2 **(B)**, and A3 **(C)**. The orange line represents the regression line, and the corresponding formula is expressed on each graph. mmHg, millimeters of mercury; *n* = 20 **(A)** and 21 **(B, C)**.

For A1, the correlation did not reach significance (*r*_*s*_ = −0.371 and p = 0.054, Figure 4A), while there was a moderate negative correlation for A2 (*r*_*s*_ = −0.500 and p = 0.010; Figure 4B) and for A3 (*r*_*s*_ = −0.512 and p = 0.009; Figure 4C).

### *P*_ET_CO_2_ prediction

**FIGURE 5:**
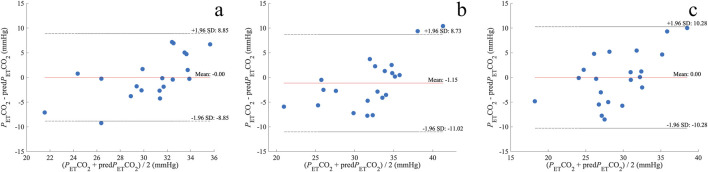
Bland–Altman plots of the difference between *P*_ET_CO_2_ and pred*P*_ET_CO_2_ before A1 **(A)**, A2 **(B)**, and A3 **(C)**. The dotted lines represent the upper limit of agreement (mean + 1.96 SD) and lower limit of agreement (mean – 1.96 SD); mmHg, millimeters of mercury; *n* = 20 **(A)** and 21 **(B, C)**.

A linear regression analysis revealed a significant predictive relationship between eMv and *P*_ET_CO_2_ in A1 (*F* (1, 18) = 6.629 and p = 0.019; Figure 4A), A2 (*F* (1, 19) = 13.994 and p = 0.001; Figure 4B), and A3 (*F* (1, 19) = 9.600 and p = 0.006; Figure 4C). eMv accounted for 27% of the explained variability in A1, 42% in A2, and 34% in A3. The linear regression equation for every apnea (Figure 4) was used to calculate the predicted *P*_ET_CO_2_ (pred*P*_ET_CO_2_) before the three apneas (Table 1). The difference between *P*_ET_CO_2_ and pred*P*_ET_CO_2_ was −0.00 ± 4.5 mmHg for A1, −1.15 ± 5.0 mmHg for A2, and −0.00 ± 5.2 mmHg for A3 (Figure 5).

### Underwater respiratory signal

**FIGURE 6:**
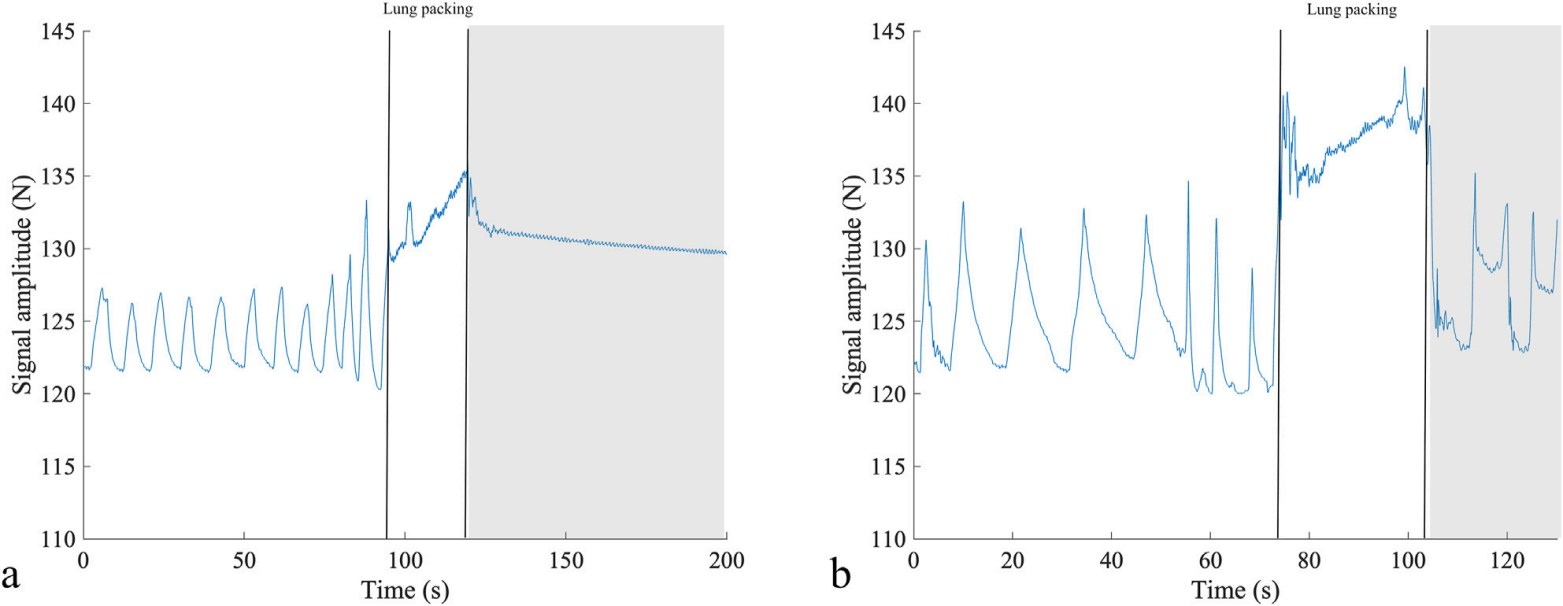
Respiratory signal from two participants before competition performance in static apnea **(A)** and dynamic apnea without fins **(B)**, depicting the differences in the breathing pattern as the diver in **(A)** shows shallower breaths but at an increased breathing frequency compared to diver **(B)**. The two vertical lines show the period of lung packing, and the gray rectangle shows the beginning of the apneic performance. N, newton; s, seconds.

The quality of the respiratory signal recorded in water before starting apneic performance was satisfactory (Figure 6).

## Discussion

Our results indicate that hyperventilation before breath-holding may be estimated using the signal from the force sensor. However, our method underestimates *P*_ET_CO_2_ values when mean values exceed 35 mmHg. Measurements appear more reliable when *P*_ET_CO_2_ is in the hypocapnic range of 25–35 mmHg, with reduced accuracy as *P*_ET_CO_2_ approaches normocapnia. These findings suggest that the prediction is more appropriate for mild hypocapnia but may be less reliable during normocapnia.

The successful application of the device for underwater performance, with good signal quality, is promising for future development. Although swimming motions, arm and leg movements, and chest compression at depth could affect the quality of the signal, the force sensor has the potential to identify involuntary breathing movements that signal the physiological breaking point (Agostoni, 1963).

### Hyperventilation

We also found that freedivers hyperventilate without noticing as they keep RR within normal or even in the lower range of normal values, which explains the previous observations in our group (unpublished work). The hyperventilation is, thus, solely due to increasing Vt. This emphasizes the challenge of quantifying the depth of breathing, a parameter that is less easily observed than RR both by the diver and observer. Quantifying the extent of hyperventilation is critical as severe hypocapnia correlates with reduced cerebral blood flow. In healthy participants, a 31% decrease in cerebral blood flow at a PaCO_2_ level of 26 ± 2 mmHg has been reported (Fortune et al., 1995). Even moderate hyperventilation can cause a 20% reduction in brain blood flow (Reivich, 1964). Additionally, patients with brain hypertension also demonstrated up to a 34% increase in brain tissue hypoxia when *P*_ET_CO_2_ values fell below 25 mmHg (Carrera et al., 2010). Some participants in our study, particularly before the third apnea, experienced severe hypocapnia (*P*E_T_CO_2_ ≤ 25 mmHg), and divers who initiate a dive with severe hypocapnia could be at a higher risk of BO. Hyperventilation alone could be a contributing factor to a transient loss of consciousness (Immink et al., 2014). At present, the relationship between the severity of hypocapnia and BO remains unclear.

### Estimating lung volumes

During quiet breathing, Vt is mainly determined by the diaphragm contraction, which induces small changes in the vertical volume of the lung (West and Luks, 2021). As the force sensor detects changes in the circumference of the thorax, it is expected to be less sensitive at lower Vt, such as in A1. However, hyperventilation typically entails a more pronounced movement of the diaphragm and accessory muscles. Consequently, this amplifies the thoracic diameter, thereby enhancing the potential to detect an increase in chest circumference using the force sensor. In our study, the estimated Vt constituted nearly 58% of the VC. We acknowledge the limitation at low volumes, which explains why we cannot estimate VC or Vt with 100% accuracy and why the correlation with *P*_ET_CO_2_ was not significant in A1. This limitation applies to all the techniques used to estimate Vt from wearables as estimating it based on the movements of the chest wall is challenging (Monaco and Stefanini, 2021). As our intention is not to use it in a clinical setting but to monitor athletes for high respiratory activity, we consider that our results are suitable for exploring practical applications in different freediving situations, including saltwater and depth. This study acts as a proof-of-concept for applying breath analysis to estimate *P*_ET_CO_2_ levels during various underwater performances.

Additionally, the respiratory signal proved instrumental in detecting thoracic changes associated with “lung packing”—a maneuver employed by freedivers to enhance their total lung capacity (Örnhagen et al., 1998; Figure 6). This maneuver was initially described as glossopharyngeal breathing in post-polio patients (Dail et al., 1955).

### Limitations

Our results apply only to dry static apneas in the sitting position, so the device should be further tested in underwater scenarios.

Additionally, despite most of the measurements being within the limits of agreement, there was a tendency to underpredict *P*_ET_CO_2_ when it was close to normal values. This means that during normal ventilation, the changes in the thoracic circumference were small and did not exert enough force in the sensor, so the amplitude of the signal was lower than expected. As we measured the changes in chest circumference in only one place, we could have missed information when ventilation was shallow or was only affecting the upper part of the chest.

## Conclusion

This study demonstrates the potential of using a force sensor to estimate hyperventilation before breath-holding under static conditions, providing a foundation for further exploration. While the prediction model accounts for a moderate proportion of the variability in *P*_ET_CO_2_, additional validation is required to establish its utility in preventive applications. Freedivers may hyperventilate even at seemingly regular or reduced breathing frequencies, emphasizing the importance of refining this approach. Further research, including underwater assessments, is essential to evaluate the feasibility of this system for improving safety in freediving.

## Data Availability

The raw data supporting the conclusions of this article will be made available by the authors, without undue reservation.

## Abbreviations

BO, blackout; eMv, estimated minute ventilation.
