# Supplementary material for: Toward a hyperventilation detection system in freediving: a proof of concept using force sensor technology
Source: Front Physiol. 2025 Jan 6;15:1498399. doi: 10.3389/fphys.2024.1498399 (PMC11743614; doi:10.3389/fphys.2024.1498399)
Supplement: Supplementary file 1 [file DataSheet1.pdf]

Supplementary Material

Vital capacity estimation

The procedure had the purpose of testing the accuracy of the force sensor to estimate the vital capacity (VC). It included 10 adult participants (4 females and 6 males) with a mean ± SD age 39 ± 10 years, height 177 ± 9 cm and weight 77 ± 8 kg. All participants were recruited by convenience sampling. They conducted a series of six slow vital capacity (SVC) maneuvers, allowing for normal breathing intervals in between. The measurements of SVC were recorded using a spirometer (Compact Expert, Vitalograph, Buckingham, UK) while the participants were in a seated position.

VC estimation results

The VC did not change across the tests (p = 0.051; Table S1) nor the amplitude in the signal from the force sensor (p = 0.123; Table S1). The VC was 4.6 ± 1.1 L, and the amplitude was 6.7 ± 1.8 N.

Table S1. Values of VC and registered amplitude by the force sensor during six tests.

	**Test 1**	**Test 2**	**Test 3**	**Test 4**	**Test 5**	**Test 6**
Vital capacity (L)	4.3 ± 1.1	4.5 ± 1.1	4.5 ± 1.1	4.6 ± 1.1	4.7 ± 1.0	4.7 ± 1.1
Amplitude (N)	6.5 ± 2.0	6.7 ± 2.0	6.6 ± 1.7	6.7 ± 1.5	7.2 ± 1.8	6.7 ± 2.0

L, liters; N, Newton

There was a strong correlation between variables $r_s = 0.712$, p = 0.021. The equation for predicted VC was = (0.4396 x Amplitude) + 1.6 (Figure S1a). The difference between measured VC and the predicted VC was 0.00 ± 0.7 L (Figure S1b).

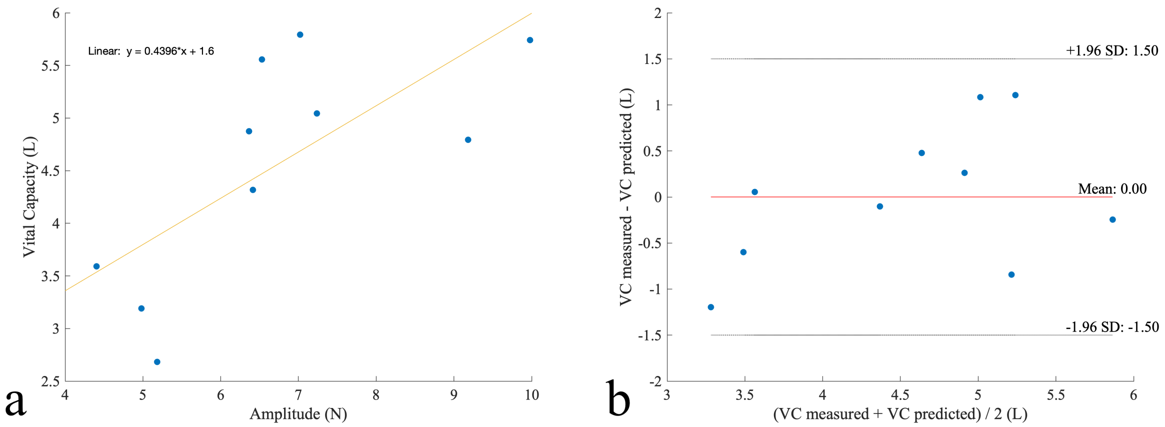

Figure S1. Comparison between VC and amplitude values from the force sensor (a), with yellow line showing the regression line and formula. Bland-Altman plot of the difference between VC and predicted VC (b), the dotted lines represent the upper limit of agreement (mean + 1.96 SD) and lower limit of agreement (mean – 1.96 SD); L, liters; n = 10
